# Comprehensive corrective exercise program improves alignment, muscle activation and movement pattern of men with upper crossed syndrome: randomized controlled trial

**DOI:** 10.1038/s41598-020-77571-4

**Published:** 2020-11-26

**Authors:** Foad Seidi, Mohammad Bayattork, Hooman Minoonejad, Lars Louis Andersen, Phil Page

**Affiliations:** 1 Health and Sports Medicine Department, Faculty of Physical Education and Sport Sciences, University of Tehran, Tehran, Iran; 2 Sport Sciences and Physical Education, Faculty of Humanities Science, University of Hormozgan, Bandar Abbas, Iran; 3 National Research Centre for the Working Environment, Copenhagen, Denmark; 4 Sport Sciences, Department of Health Science and Technology, Aalborg University, 9220 Aalborg, Denmark; 5 Franciscan University DPT Program, Baton Rouge, LA USA

**Keywords:** Anatomy, Health care, Medical research

## Abstract

Upper crossed syndrome (UCS) refers to the altered muscle activations and movement patterns in scapulae along with some abnormal alignment in the upper quarter, which may contribute to the dysfunction of the cervicothoracic and glenohumeral joints. The present study aimed to investigate the effectiveness of a comprehensive corrective exercise program (CCEP) and subsequent detraining on alignment, muscle activation, and movement pattern in men with the UCS. This randomized controlled trial included 24 men. The intervention group conducted CCEP (8 weeks), followed by four weeks of detraining and the control group maintained normal daily activities. Electromyography of selected muscles, scapular dyskinesis test, head, shoulder, and thoracic spine angle were measured at baseline, post-test, and follow-up. There were significant differences for Group x time interaction and also for within-group from pre-test to post-test and follow-up in all outcomes. Also, significant differences were observed in three outcomes at post-test and follow-up between the CCEP and control group in favor of the CCEP. In Conclusion, the present study demonstrates that the CCEP for individuals with UCS is feasible and effective, improving muscle activation imbalance, movement patterns, and alignment. Importantly, these improvements were maintained after four weeks of detraining, suggesting lasting neuromuscular re-training adaptations.

## Introduction

Proper posture is important for optimal functional performance, and is associated with many biomechanical, motor control, and performance variables^1,2^. Deviation from healthy posture suggests the presence of neuromuscular imbalance and may be associated with certain musculoskeletal disorders^3–5^. Upper crossed syndrome (UCS) is an abnormal posture that according to Vladimir Janda (1923–2002) refers to a specifically altered muscle activation pattern (especially in the neck, trunk and scapular muscles) and altered movement patterns (scapular dyskinesis) along with postural deviations (forward head and shoulder posture, and increased thoracic kyphosis)^6,7^. These changes can lead to various musculoskeletal symptoms in the head, neck, shoulder, and upper back^7–9^, and it is, therefore, essential to quantify UCS behavior because of its consequences.

In an attempt to correct abnormal postures, clinicians and therapists have designed exercises based on biomechanical and neurological approaches^10–13^. Although these approaches seem to work in clinical practice, there are some limitations. Review studies have questioned the effectiveness of exercise programs based on the biomechanical approach, and the neurological approach has not been tested in practice to improve the postural malalignments^14–16^. Furthermore, high-quality randomized studies are needed in this field of research.

The Comprehensive Corrective Exercise Program (CCEP) is based on a new approach (Comprehensive Approach), which is designed to seek innovation by using advantage of the strengths and weaknesses of previous approaches^17,18^. Indeed, the comprehensive approach is based on the system view, which in the interaction between different parts of a system is responsible for providing important information about the overall performance and behavior of the system^19^. In a complex system such as the human movement system, there is an interaction between the articular, muscular, and neural subsystems in the production of movement^6,20^. It is, therefore, imperative that in evaluating and correcting musculoskeletal problems, like UCS, the interactions between these subsystems that ultimately provide system performance and overall behavior should be considered^17,19^.

Moreover, the UCS could be a sign of underlying potential sensorimotor dysfunction, which leads to an imbalance in muscle activation, movement pattern, and postural alignment^7,21^. Therefore, the CCEP can be useful in improving UCS because of a multifaceted focus on muscle activation, movement pattern, and posture simultaneously across the whole body rather than focusing only on the part of the body where the pain occurs^17,18^. While most of the previous studies have only focused on the assessment and correction of postural aspects of the UCS, researchers have only evaluated the alignment of the neck or upper back before and after an exercise program in which have only used stretching/strengthening training^12,22,23^. Therefore, to get the optimal correction of the UCS, the exercise program should emphasize not only biomechanical but also neurological factors.

The comprehensive approach is novel in the field of corrective exercises designed to correct musculoskeletal disorders and to prevent secondary complications such as pain and injury^18^. Therefore, extensive testing is needed, including quantifying malalignments such as UCS with many secondary musculoskeletal changes and complications of high prevalence in sedentary individuals, especially those in poor postural conditions^7–9^. Furthermore, none of the previous studies have investigated whether changes are maintained following a period of detraining which is highly relevant to assess whether lasting effects occur.

The primary aim of the present study was to evaluate the effectiveness of CCEP in young men with the UCS, as measured by alignment (head and neck, shoulder and thoracic spine), the electromyography activity of selected muscles (upper, middle, lower trapezius, and serratus anterior), and specific movement patterns (scapular dyskinesis test). The secondary aim was to quantify maintenance following detraining.

## Results

Table 1 shows the baseline demographic and all variable characteristics for each group. There was no significant difference between the two groups for any of demographic and outcome variables. Although some outcome variables varied between CCEP and control groups, they were not significantly different. Repeated-measures ANOVAs were used to compare alignment, muscle activation, and movement pattern of UCS subjects at both post-test and follow-up between the CCEP and control groups (Table 2). There was a significant group by time interaction (p < 0.05) for each outcome (except the onset of muscle activities); that is, the CCEP and control groups changed differently over time.

**Table 1:** Baseline demographics characteristics in all study groups.

Variables	CCEP	Control group	Comparison^a^
Age (year)	25.3 ± 2.5	25.4 ± 1.5	t = 0.907, p = 0.81
Height (cm)	176.8 ± 7.2	179.1 ± 3.5	t = 0.225, p = 0.44
Weight (kg)	77.7 ± 2.5	75.7 ± 3.9	t = 0.932, p = 0.58
BMI (kg/m^2^)	23.8 ± 0.72	23.78 ± 0.9	t = 0.99, p = 0.31

^a^Comparison: by t-test for age, height, weight and BMI variables.

**Table 2:** Within-group differences in alignment, muscle activation, and movement pattern in the CCEP and control group, P < 0.05.

Outcomes measures	CCEP group					Control group				
	Pre-test	Post-test	Follow-up	f	*P-value*	Pre-test	Post-test	Follow-up	f	*P-value*
**Alignment (degree)**										
FHA	46.71 ± 2.39	39.52 ± 1.96	40.57 ± 2.03	25.176	0.002*	47.12 ± 1.82	48.01 ± 2.04	48.27 ± 1.08	13.425	0.391
FSA	54.36 ± 2.22	45.45 ± 1.87	46.46 ± 1.02	8.936	0.001*	53.79 ± 2.13	52.81 ± 2.52	53.12 ± 1.87	14.822	0.539
TKA	47.90 ± 2.56	36.34 ± 1.85	38.17 ± 1.21	32.385	0.001*	46.73 ± 1.84	47.26 ± 2.05	47.15 ± 2.29	9.207	0.278
**Muscle activation (%MVIC)**										
UT (Conc)	26.28 ± 10.97	16.40 ± 8.09	15.76 ± 7.81	48.105	0.001*	21.10 ± 5.43	21.94 ± 5.90	23.84 ± 8.57	3.496	0.041*
UT (Iso)	21.72 ± 6.90	17.48 ± 7.09	18.32 ± 7.32	17.593	0.001*	18.82 ± 3.64	22.11 ± 5.24	25.16 ± 7.31	7.794	0.003*
UT (Ecc)	19.11 ± 4.93	13.05 ± 4.83	12.59 ± 5.48	42.041	0.001*	12.90 ± 4.32	16.39 ± 5.31	19.24 ± 5.36	17.490	0.001*
MT (Conc)	15.69 ± 6.34	26.74 ± 10.13	21.66 ± 9.31	23.271	0.001*	18.95 ± 4.46	16.16 ± 6.30	15.07 ± 4.91	4.025	0.073
MT (Iso)	8.32 ± 4.05	16.62 ± 4.72	14.64 ± 5.32	90.515	0.001*	11.60 ± 5.89	9.01 ± 3.98	8.70 ± 3.88	4.273	0.056
MT (Ecc)	10.66 ± 2.66	17.15 ± 4.39	12.94 ± 2.32	15.788	0.001*	14.54 ± 4.76	12.16 ± 3.01	11.21 ± 3.48	4.022	0.065
LT (Conc)	11.28 ± 4.67	20.71 ± 5.18	17.70 ± 5.15	63.389	0.001*	18.95 ± 4.46	14.39 ± 5.30	13.07 ± 5.31	14.305	0.041*
LT (Iso)	12.76 ± 4.17	20.02 ± 3.55	19.29 ± 4.87	30.246	0.001*	27.60 ± 5.89	19.10 ± 7.98	17.08 ± 8.66	18.881	0.018*
LT (Ecc)	11.76 ± 4.56	22.28 ± 6.67	17.93 ± 4.85	49.606	0.001*	21.54 ± 4.76	16.39 ± 8.19	14.89 ± 7.79	11.829	0.032*
SA (Conc)	17.38 ± 6.79	25.55 ± 6.91	23.47 ± 6.60	65.156	0.001*	26.11 ± 11.50	15.31 ± 5.13	13.47 ± 4.05	11.405	0.006*
SA (Iso)	19.52 ± 10.28	31.87 ± 13.77	29.28 ± 14.2	27.156	0.001*	26.49 ± 9.27	16.88 ± 6.12	16.82 ± 6.23	25.176	0.011*
SA (Ecc)	12.60 ± 3.06	21.68 ± 5.55	19.27 ± 3.85	30.729	0.001*	16.13 ± 6.89	8.96 ± 3.35	8.75 ± 2.89	9.957	0.034*
UT/MT (Conc)	1.96 ± 0.91	0.96 ± 0.66	1.06 ± 0.68	33.776	0.001*	2.31 ± 1.55	2.75 ± 1.51	2.86 ± 1.47	10.244	0.071
UT/MT (Iso)	1.96 ± 0.78	0.81 ± 0.44	1.02 ± 0.37	26.706	0.001*	3.45 ± 1.98	3.83 ± 1.79	3.83 ± 1.94	2.414	0.193
UT/MT (Ecc)	2.20 ± 1.40	1.10 ± 0.64	1.38 ± 0.79	10.463	0.007*	2.33 ± 1.59	3.08 ± 1.17	3.39 ± 1.26	13.350	0.041*
UT/LT (Conc)	2.01 ± 0.90	1.04 ± 0.53	1.05 ± 0.56	26.076	0.001*	1.93 ± 0.94	2.26 ± 1.01	2.35 ± 1.15	1.749	0.201
UT/LT (Iso)	1.86 ± 0.46	0.98 ± 0.35	1.13 ± 0.51	28.362	0.001*	1.50 ± 0.88	1.88 ± 0.89	2.09 ± 0.97	9.4249.424	0.031*
UT/LT (Ecc)	2.70 ± 1.02	1.58 ± 0.77	1.66 ± 1.11	43.311	0.001*	1.65 ± 0.94	2.50 ± 0.88	2.23 ± 1.24	9.051	0.027*
UT/SA (Conc)	1.41 ± 0.85	0.65 ± 0.38	0.78 ± 0.44	18.062	0.001*	2.16 ± 1.09	2.89 ± 1.54	2.90 ± 1.42	5.486	0.081
UT/SA (Iso)	1.92 ± 1.33	0.64 ± 0.32	1.14 ± 0.77	12.862	0.004*	1.78 ± 1.00	2.01 ± 1.09	2.04 ± 1.12	11.431	0.063
UT/SA (Ecc)	1.80 ± 1.22	1.18 ± 0.78	1.13 ± 0.83	43.311	0.001*	2.70 ± 1.60	3.06 ± 1.50	3.21 ± 1.48	9.051	0.141
Onset (UT)	− 0.20 ± 0.88	0.24 ± 0.34	0.06 ± 0.22	2.889	0.068	− 0.01 ± 0.90	0.20 ± 0.43	0.18 ± 0.43	2.889	0.068
Onset (MT)	− 0.13 ± 0.65	− 0.05 ± 0.46	0.01 ± 0.35	0.507	0.607	− 0.11 ± 0.41	0.06 ± 0.31	− 0.09 ± 0.38	0.507	0.607
Onset (LT)	− 0.12 ± 0.43	0.19 ± 0.63	0.24 ± 0.66	1.774	0.183	− 0.11 ± 0.41	0.09 ± 0.22	− 0.21 ± 0.50	1.774	0.183
Onset (SA)	0.26 ± 0.44	0.12 ± 0.44	0.06 ± 0.39	3.607	0.061	0.13 ± 0.35	0.21 ± 0.32	0.07 ± 0.36	3.607	0.061
**Movement pattern**										
SDT	2.48 ± 0.29	5.15 ± 0.21	4.47 ± 0.19	15.263	0.001*	2.31 ± 0.53	2.17 ± 0.41	2.27 ± 0.35	1.548	0.523

### Within-group comparison for the CCEP and control group

Table 2 shows that there were significantly different (p < 0.05) for all three outcomes (alignment, muscle activation, and movement pattern) in the CCEP from pre-test to post-test and follow-up. In this regards, the alignment outcomes (including FHA, FSA, TKA) and some muscle activation outcomes (including UT, UT/MT, UT/LT, UT/SA) were decreased, and movement patterns (scapular dyskinesis) and muscle activation in MT, LT, and SA were increased in the CCEP. Interestingly, there were significantly different (p < 0.05) levels of UT, LT, and SA activations in the control group from pre- to post-test and follow-up, with increasing UT activation and decreasing LT and SA activations. The onset of muscle activations did not change significantly for either group over time (Table 2).

Considering the pairwise comparisons for the participants in the CCEP group, the alignment, muscle activations (except the onset), and movement patterns significantly improved from pre-test to both post-test and follow-up (p < 0.05). However, the same changes were not statistically significant from post-test to follow-up (between the end of the program training and after 4-weeks detraining) (Table 2).

### Between-group comparison at posttest and follow-up

Significant differences were observed between the CCEP and control group in all three outcomes (alignment, muscle activation, and movement pattern) at the post-test and follow-up to the favor of the CCEP (Table 3). No significant differences were noted concerning the onset of muscle activations.

**Table 3:** Between-group differences in alignment, muscle activation, and movement pattern in the CCEP and control group, P < 0.05.

Outcomes measures	Post-test			Follow-up	
	*f*	*P*-value	Effect size	*f*	*P*-value
**Alignment**					
FHA	16.957	0.003*	0.489	18.263	0.001*
FSA	34.591	0.001*	0.367	25.461	0.001*
TKA	28.071	0.001*	0.691	17.298	0.007*
**Muscle activation**					
UT (Conc)	40.496	0.001*	0.779	21.495	0.001*
UT (Iso)	15.88	0.001*	0.498	14.605	0.001*
UT (Ecc)	37.905	0.001*	0.703	35.499	0.001*
MT (Conc)	18.272	0.001*	0.549	18.791	0.001*
MT (Iso)	37.251	0.001*	0.713	29.883	0.001*
MT (Ecc)	10.723	0.005*	0.417	11.747	0.008*
LT (Conc)	26.962	0.001*	0.643	18.245	0.001*
LT (Iso)	15.036	0.001*	0.501	11.012	0.004*
LT (Ecc)	54.762	0.001*	0.785	30.073	0.001*
SA (Conc)	36.547	0.001*	0.709	28.154	0.001*
SA (Iso)	16.041	0.001*	0.517	29.728	0.001*
SA (Ecc)	34.031	0.001*	0.694	49.943	0.001*
UT/MT (Conc)	63.716	0.001*	0.780	52.064	0.001*
UT/MT (Iso)	28.055	0.001*	0.609	41.362	0.008*
UT/MT (Ecc)	64.723	0.001*	0.782	60.649	0.001*
UT/LT (Conc)	36.233	0.001*	0.668	43.638	0.001*
UT/LT (Iso)	35.561	0.001*	0.664	39.741	0.001*
UT/LT (Ecc)	36.890	0.001*	0.672	48.783	0.001*
T/SA (Conc)	17.355	0.001*	0.491	15.846	0.001*
UT/SA (Iso)	35.749	0.001*	0.665	21.630	0.001*
UT/SA (Ecc)	28.867	0.001*	0.616	26.954	
Onset (UT)	0.443	0.514	0.003		
Onset (MT)	0.005	0.947	0.002		
Onset (LT)	1.576	0.225	0.020		
Onset (SA)	1.538	0.230	0.001		
**Movement pattern**					
SDT	8.285	0.001*	0.669	11.263	0.001*

### Effect size and MCID results

**Figure 1:**
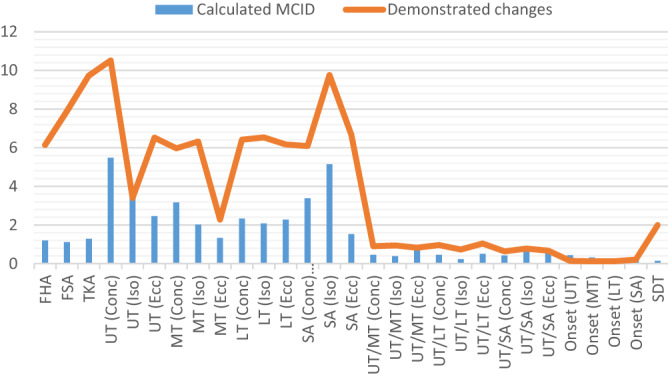
The results of MCID in the CCEP group.

The result showed that the CCEP group demonstrated a large effect size (η^2^ ≥ 0.14) improvement in all three outcomes, including alignment, muscle activation (except the onset), and movement pattern at the post-test when compared to the control group. Also, the demonstrated changes in the outcomes from baseline to follow-up were more than MCIDs that were calculated by the mentioned formula (Fig. 1).

### The rates of attendance to the CCEP

Mean attendance to the CCEP was 89.6 ± 1.4% (range 83.3–100%) of all possible sessions with no dropouts.

## Discussion

This study evaluated the effects of the CCEP compared with a control condition on alignment, muscle activation patterns of the main scapular stabilizers, and related movement patterns among young men with the UCS. Mean EMG amplitude and onset of the UT, MT, LT, and SA, as well as alignment factors including FHA, FSA, and TKA, along with related movement pattern (scapular dyskinesis), were measured for both the CCEP and control groups at baseline (pre-test), week 8 (post-test), and week 12 (follow-up). The research hypothesis was that the CCEP would significantly improve the selected muscle activation, movement patterns, and alignment of the head, shoulder, and thoracic spine. We also hypothesized that the effects following the CCEP would be maintained after four weeks of detraining.

According to our results, the CCEP used in this study appears to have an acceptable effect on restoring balance in the scapula stabilizer muscles. This is one of the main findings of the present study because, according to the chain reactions expressed by Janda, the scapula is considered as the key-stone and source of complications in the UCS^7^. It is plausible that one of the main reasons for the restoring muscle activity of the scapular stabilizer following the implementation of the CCEP was the emphasis on cognition as a part of neuromuscular rehabilitation, especially in the initial phase. The role of neuromuscular rehabilitation is to change movement patterns or motor behavior. Furthermore, cognition can modify or facilitate motor behavior and control^24^; therefore, having chronic musculoskeletal pain patients mentally focus on muscle contraction during corrective exercises may be beneficial. To utilize these cognitive benefits, the participants in the CCEP group used an internal focus of attention to contract underactive scapular muscles or to relax overactive muscles for normalization of scapular position and, if needed, the therapist gave verbal or tactile feedback. Researchers in the field of scapular rehabilitation have previously described this type of exercise as “scapular orientation exercises”^25^. Mottram et al. showed that individuals could be trained to position the scapula in upward rotation and posterior tilt by creating appropriate contractions in the three parts of the trapezius muscle^25,26^.

Restoring motor control and balance of scapular muscle activations are more critical than increasing strength in these muscles^27^. Therefore, some researchers have used EMG biofeedback to learn the correct contractions of the scapular muscles during neuromuscular exercises. These previous studies showed that conscious exercises with feedback have immediate effects on controlling movement and kinematics of the scapulae^27,28^. Holterman et al. showed that after one hour of biofeedback by EMG, all subjects learned to activate parts of the trapezius muscle (such as the lower part) while simultaneously relaxing the other part (upper part)^29^. Moreover, researchers have shown that musculoskeletal disorders can be associated with reorganization of the cerebral cortex^30^. Therefore, retraining muscle activity using motor learning principles and neuromuscular function can restore proper muscle application patterns in the early stages of the training program^24,29^.

At the end of the initial phase of CCEP, the participants gradually gained the ability to create concentric and eccentric contractions while performing the movement in different positions of the exercise. The logic of exercise progression (from isometric to dynamic) has been confirmed in previous studies^31^. Our results showed an improvement in selected muscle activations in all three phases of concentric, isometric, and eccentric, which can be due to training in all contraction phases.

Another reason for the improvement in selected muscle activations was probably related to the use of targeted exercises in CCEP for scapular dyskinesis rehabilitation, which increased the activity of the MT, LT, and SA and reduced the activity of the UT^32^. All of these exercises have been mentioned as exercises that can create the preferred activation ratio between the scapular stabilizer muscles^33,34^. The present study showed a clinically-desired, significant decrease in the ratio of the UT/MT, UT/LT, and UT/SA after the end of the CCEP.

Although there was no significant change in the onset of muscle activation following the CCEP, our study suggests the timing of muscle activation seems to be closer to normal. The timing of muscle activation is an essential factor in the coordination between the scapula and arm movement^35,36^. The timing of the experimental group before performing CCEP was as follows: first, the UT (− 0.20 ms), then the MT (− 0.13 ms), the LT (− 0.12 ms), and then the SA (0.26 ms) was activated. Theoretically, the middle and lower parts of the trapezius muscle play a more stabilizing role; the delay in their activation compared to the UT, as seen in this study, can lead to a relative dominance of the UT^35^. This relative dominance at the onset, along with the higher level of activity of the UT than the MT, LT, and SA, creates muscle imbalance around the scapula, and ultimately leads to dysfunction in the rhythm and movement of the scapula (scapular dyskinesis)^21,37^. The results showed that despite the lack of significant differences, the timing of scapular muscle activations was changed after CCEP as following: first, the MT (0.05 ms), the SA (0.12 ms), the LT (0.19 ms), and finally the UT (0.24 ms) were activated. This could indicate that the upper part of the trapezius muscle was not superior to other parts after performing CCEP.

Our results demonstrated that the CCEP can improve the movement patterns (scapular dyskinesis) and the alignments of the head, shoulder, and thoracic spine in people with UCS. It seems that improving in scapular dyskinesis and alignment would be followed by improvement in neuromuscular factors created by cognition and conscious control of experimental participants after CCEP. Previous evidence has shown that people with scapular dyskinesis can obtain a proper position and movement of the scapula by consciously controlling the scapula^26,38^. Cools et al. also noted the importance of the correct alignment of the head and spine during scapular rehabilitation exercises^39^; the authors stated that this strategy of simultaneous correction of the posture should be noted in all phases of the rehabilitation program. After using internal focus of attention and regaining sufficient control over scapular muscles in the CCSP, participants then focused externally on correcting related segments through chin tuck, retraction of shoulders, and straightening the upper thoracic spine^18^. We believe the improvement in postural deviations and scapular position and rhythm in the CCEP group was due to the interaction of improved muscle activity, movement pattern and alignment.

For the secondary purpose of the current study, the results showed that the positive effects following the CCEP were maintained after four weeks of detraining. The present study is the first we are aware of to investigate the effect of a short-term detraining on the scapula stabilizer muscle activations and movement patterns, as well as related postural deviations after applying an 8-week training intervention. Previous research suggests there should be an increase in muscle function at the beginning of an exercise program related to physiological and neurological adaptations. Prior to hypertrophic gains, early muscular adaptations to resistance training include applying more motor units, learning more effective and economical use of active motor units, and reducing inhibitory inputs for alpha motor neurons^40,41^. Optimal neuromuscular changes in the initial and improvement phases of CCEP followed by maintenance of these changes after a month of detraining support the lasting positive effects of corrective exercise on muscle activations, movement patterns and alignment in participants with UCS.

Our study had some limitations, including the recruitment of only young males; therefore, the results of this study may not be generalizable to all groups (e.g., women or men aged ≥ 28 years) with the UCS. Also, the study was performed on a relatively small sample size; however, the effect sizes of the differences in outcome measures between the CCEP and control groups suggest that the findings are less likely to be affected by sample size. Another limitation is the lack of a double-blind design, which is not feasible with exercise interventions.

## Conclusion

This study demonstrated that the CCEP for individuals with UCS is feasible and results in improvement of muscle imbalance, movement patterns, and postural alignment that are maintained after short-time detraining. Therefore, our approach to improving the UCS could represent a fundamental paradigm shift in exercise intervention strategies to improve postural malalignments and their consequences. This study may assist practitioners in individualized clinical decision-making; however, our results may have a limited generalizability to all individuals with UCS.

## Methods

### Study design

The current study was a parallel-group randomized, controlled trial comparing the 8-week CCEP, followed by four weeks of detraining to a control group without any intervention. The study has been registered at the Iranian Registry of Clinical Trials on 26 October 2018 (IRCT20181004041232N1), and the ethical approval was obtained by the Ethics Committee on Research at the University of Tehran, Iran (IR.UT.REC.1395026). The study was conducted at the Laboratory of Health and Sports Medicine Department, University of Tehran, Tehran, Iran. The study was reported in accordance with the rigor of the CONSORT guideline, and all experimental conditions conformed to the Declaration of Helsinki. The study protocol has been published elsewhere^18^.

### Participants

The participants consisted of 24 men aged 18 to 28 years with the UCS. The process of recruiting and screening is reported elsewhere^18^. All participants completed and signed the informed consent form. The inclusion criteria were having any abnormality in the position and rhythm of the scapula, as measured by the scapular dyskinesis test, having postural changes such as excessive thoracic kyphosis (≥ 42°), forward head (≥ 44°) or round shoulder (≥ 49°) as measured by flexicurve and photogrammetry, respectively^17^. Exclusion criteria were having any visible malalignment in the pelvis or lower extremities, have a rotation higher than 5 degrees on the forward bending test because of scoliosis, which was measured with a scoliometer^17^, have a history of joint diseases in the spine, shoulder, and pelvis, fracture, surgery, and have a bodyweight outside the normal range (BMI between 18 and 25)^42^.

### Randomization

**Figure 2:**
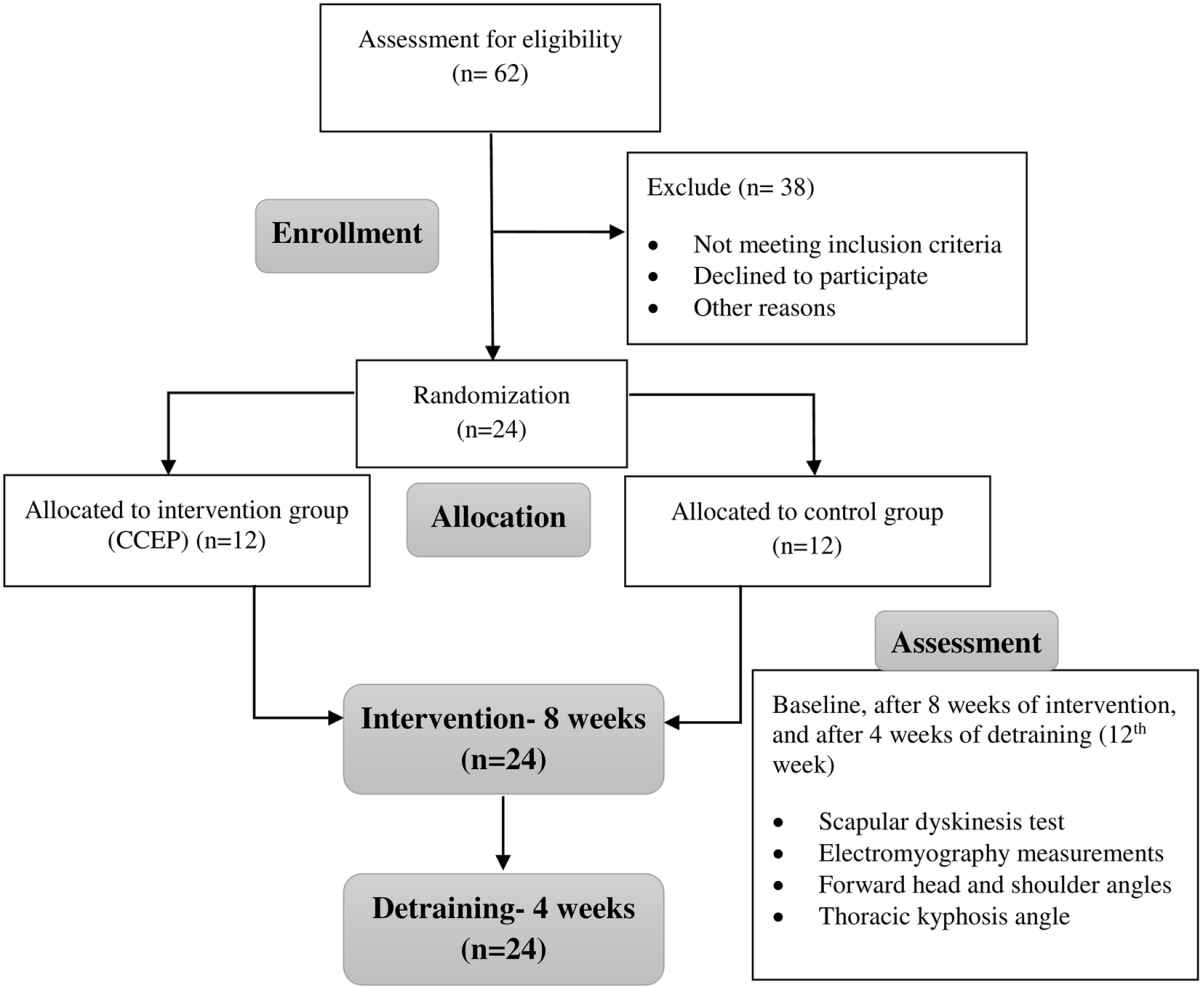
Study flowchart.

Participants were randomized using computer-generated block randomization in a 1:1 ratio, followed by a concealed allocation through opening the sequentially numbered, opaque and sealed envelopes, and a card inside indicated the group into which the participant was randomly allocated, i.e., the intervention or the control group. After randomization, participants took part in the baseline assessment process, and then the treatment group received the interventions for eight weeks, while the control group did not receive any intervention. All the measurements were repeated after ending the intervention. Finally, a follow-up assessment was performed after a 4-week detraining period. The study flowchart is shown in Fig. 2.

### Intervention

The duration of the exercise protocol was eight weeks and three sessions per week, and each session was about an hour. Each exercise session began with 10 min of warm-up activity, ended with 5 min of cool-down, and all exercises were performed under supervision. The participants did not conduct any extra exercises at home; however, it was essential to avoid sustaining poor postures. The control group did their ordinary daily activities and did not participate in any exercise programs. After the study was completed, the control group received the exercise intervention protocol for ethical considerations. The specific intervention protocol has been described in detail elsewhere^18^ and is briefly summarized below. Although there was a framework for the CCEP, shown in Table 4, exercises were progressed by considering individual characteristics of each participant.

**Table 4:** Comprehensive corrective exercise program framework.

Exercise	Intensity/duration	Equipment	Exercise	Intensity/duration	Equipment
Initial Phase (2 weeks)			Improvement Phase (5 weeks)		
Exercise 1A-C	From10s hold × 7 to 15 s hold × 10	Roller	Exercise 6	From 10 repetitions × 5 to 15 repetitions × 6	Dumbbell
Exercise 2	From10s hold × 7 to 15 s hold × 10	–	Exercise 7	From 10 repetitions × 5 to 15 repetitions × 6	Dumbbell
Exercise 3	From10s hold × 7 to 15 s hold × 10	–	Exercise 8	From 10 repetitions × 5 to 15 repetitions × 6	Dumbbell
Exercise 4	From10s hold × 7 to 15 s hold × 10	–	Exercise 9	From 10 repetitions × 5 to 15 repetitions × 6	Thera-band
Exercise 5	From10s hold × 7 to 15 s hold × 10	–	Exercise 10	From 10 repetitions × 5 to 15 repetitions × 6	Thera-band
–	–	–	Exercise 11	From 10 repetitions × 5 to 15 repetitions × 6	Swiss ball
–	–	–	Exercise 12	From 10 repetitions × 5 to 15 repetitions × 6	Swiss ball
–	–	–	Exercise 13	From 10 repetitions × 5 to 15 repetitions × 6	Balance board

#### Comprehensive Corrective Exercises Program (CCEP)

**Figure 3:**
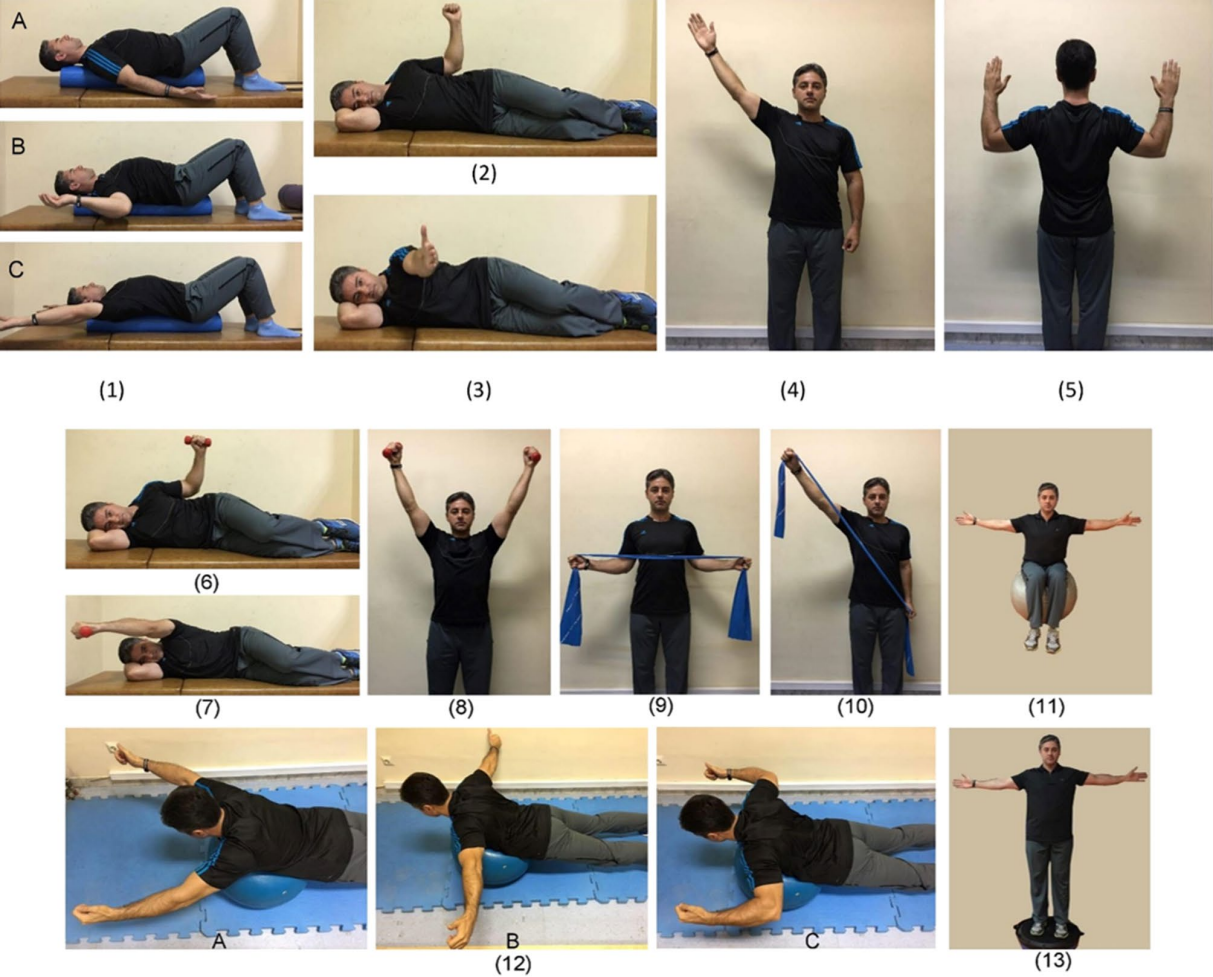
The initial phase exercises: lay supine on the foam roll in three different arm abduction angles (exercise 1A-C), side-lying external rotation (exercise 2), side-lying forward flexion (exercise 3), standing diagonal flexion (exercise 4), and military press (exercise 5). Improvement phase exercises: side-lying external rotation with dumbbell (exercise 6), side-lying forward flexion with dumbbell (exercise 7), standing diagonal flexion with dumbbell (exercise 8), standing external rotation with Thera-band (exercise 9), standing diagonal flexion with Thera-band (exercise 10), abduction in sitting on a training ball (exercise 11), lying prone V, T, and W exercises (exercise 12), and abduction in standing on the balance board (exercise 13).

The CCEP was designed in three phases, including initial, improvement, and maintenance. Exercises are progressed in frequency and intensity during these phases, as long as the movements are performed in a good quality. The exercises in the initial phase were characterized with a cognitive focus on scapular muscles (i.e., the internal focus of attention). Subjects were instructed to contract underactive muscles isometrically and relax overactive muscles for normalization of scapular position and motion^25,31^. After restoring the muscle balance in the static conditions, participants added upper extremity movements in various training positions (Fig. 3: exercises 1–5).

Once the participants could contract appropriate muscles in correct alignment during the movement pattern, the protocol focused on improving sustained postures. This goal was addressed in the improvement phase when necessary tissue adaptations occurred by increasing the load of exercises (Fig. 3: exercises 6–13)^43,44^. In the maintenance phase, the participant continued to do the exercises and maintain the training adaptations for two weeks^44^. The exercises were the same as the improvement phase without any progression in intensity and frequency.

### Outcome measures

Demographic characteristics (i.e., age, weight, height, BMI) were measured at baseline. All outcome measurements were performed by the main researcher at the baseline, eight weeks (posttest), and 12 weeks (follow-up).

#### Electromyography measurement

Surface electromyography of the selected muscles, including the upper trapezius (UT), middle trapezius (MT), lower trapezius (LT), and serratus anterior (SA) were performed using a ME-6000 Megawin (MegaWin, Finland). The participants performed humeral abduction without resistance in three phases (concentric, isometric, and eccentric) lasting for 3 s each. They had already been trained to achieved the reliable reproduction of the movement at the required velocity. A synchronized electrogoniometer and a metronome were used to control the three phases. Speed was standardized to a count of 3 s in the concentric phase, a second at full range abduction (isometric phase) and 3 s in the eccentric phase of abduction motion. Therefore, they performed the movement five times, and the rest time lasted 3 s in-betweens. Disposable Ag–AgCl electrodes with a diameter of 2 cm and a 2 cm spacing between two poles of electrodes were used, and data were recorded at a frequency of 1000 Hz. The location of the electrodes was determined using the SENIAM protocol and based on valid scientific papers^45,46^. The maximum voluntary isometric contraction (MVIC) was recorded to normalize the signals^18^. The data from the mean square root (RMS) was used in the process of measuring muscle activation. Muscle activation ratios were also calculated for the mean EMG amplitude; a ratio less than one indicates higher MT, LT, or SA activation than UT, and an amount greater than one indicates greater UT activation than MT, LT, or SA^46^. Only the concentric phase of the motion was used to determine the onset of muscle activity, and it was based on the onset of the deltoid muscle. Moreover, the onset of muscle activation was from the point where the level of muscle activity reached three standard deviations above the rest of the muscle activity^46^.

#### Scapular dyskinesis

The dynamic scapular dyskinesis test, according to the procedure described by McClure et al.^47^, was used to assess the scapular movement pattern. The position and motion of scapula were characterized by dyskinesis as a “yes” (presence of deviation or dysrhythmia/asymmetry bilaterally) or “no” (no presence). This method has been shown to be reliable among observers and has acceptable clinical utility^47,48^.

#### Forward head and shoulder angles

The forward head and shoulder angles were measured using the photogrammetry method according to the procedure described elsewhere^17,49^. The validity and reliability of this method have been established in previous studies^50,51^.

#### Thoracic kyphosis angle

The Flexicurve method was used to measure the static alignment of the thoracic spine, which is a well-established, valid, and reliable technique^52,53^. A detailed description of the procedure can be found in previous studies^17,42^.

### Statistical Method and analysis

The sample size was calculated using the G*Power software (G*Power, Version 3.0.10, Germany) and have been described in detail elsewhere^18^. Assessments of statistical procedures were performed using IBM SPSS version 20 for Windows (SPSS Inc., Chicago, IL, USA). The independent samples t-test was used to compare all outcome variables at baseline. A 2(group) × 3(time) Mixed model repeated measures ANOVA was used to compare all values from the pre-test value to each time point within each group. Analyses testing for within-group changes were also performed using mixed-model repeated-measures analysis of variance. For any significant difference, a Bonferroni post-hoc test to denote significance was used for follow-up analysis. One-way ANCOVA was used to compare groups in the post-test and follow-up with each pre-test value as a covariate. The effect size was calculated for the magnitude of the difference using the partial η^2^ method as small (0.01 ≤ η^2^ < 0.06), medium (0.06 ≤ η^2^ < 0.14) or large (η^2^ ≥ 0.14)^54,55^. Also, the following formula (MCID = SD × 0.5) was used to calculate the minimum clinically important difference (MCID) in this study^56^. The significance level was set at p < 0.05, and all data are presented as M ± SD.

### Ethics approval and consent to participate

Ethical approval was obtained on August 28, 2017, by the Ethics Committee on Research at the University of Tehran, Iran (IR.UT.REC.1395026). Before starting the project, all participants were asked to complete the written consent form.

### Consent for publication

Written informed consent was obtained from the person for publication of his accompanying images in this manuscript.
